# Story of an Unfortunate Fall: Cardiac Contusion Presenting with an Atrioventricular Block

**DOI:** 10.7759/cureus.4650

**Published:** 2019-05-13

**Authors:** Maryam Saleem, Fatima Ahmed, Kinjan Patel, Muhamad B Munir, Mary Warden

**Affiliations:** 1 Internal Medicine, West Virginia University, J.W. Ruby Memorial Hospital, Morgantown, USA; 2 Internal Medicine, Charleston Area Medical Center, Charleston, USA; 3 Cardiology, West Virginia University, J.W. Ruby Memorial Hospital, Morgantown, USA

**Keywords:** cardiac contusion, heart block, pacemaker, atrioventricular

## Abstract

Blunt cardiac injury (BCI), also referred to in the literature as a cardiac contusion, is a known cause of myocardial injury. It is often challenging to diagnose this condition in the absence of clear diagnostic criteria. Furthermore, its clinical presentation is highly variable depending on the severity, type, and duration of the trauma, as well as the timing from the initial insult. The clinical manifestation of BCI ranges from none to fatal arrhythmias to cardiac wall rupture seen on post-mortem examination. Cardiac biomarkers and electrocardiograms (EKG) are usually helpful in identifying cardiac trauma but are not necessarily abnormal in all cases.

Falls by slipping on ice are common in the winter, but rarely do people present with a myocardial injury with these mechanical events. We describe the case of a cardiac contusion with an unusual presentation and an unusual cause, whereby both the initial EKG and troponin level were normal, and the patient presented with an atrioventricular (AV) block two weeks after “slipping on ice”.

## Introduction

Trauma to the heart can lead to structural abnormalities, which are usually easier to detect, as well as conduction abnormalities that may present a diagnostic challenge for the clinician. The arrhythmias or conduction abnormalities caused by blunt cardiac injury (BCI) can be transient or persistent and may present acutely or have a delayed presentation. Damage to the atrioventricular node (AVN) can cause different degrees of atrioventricular block. Complete heart block (CHB) is a known complication of BCI and has been reported occasionally in the medical literature [1-5]. The exact mechanism remains poorly understood, especially in the setting of CHB occurring weeks after a fall, in an otherwise structurally and electrophysiologically normal heart. We present a case of CHB developing secondary to cardiac contusion and its management.

## Case presentation

**Figure 1:**
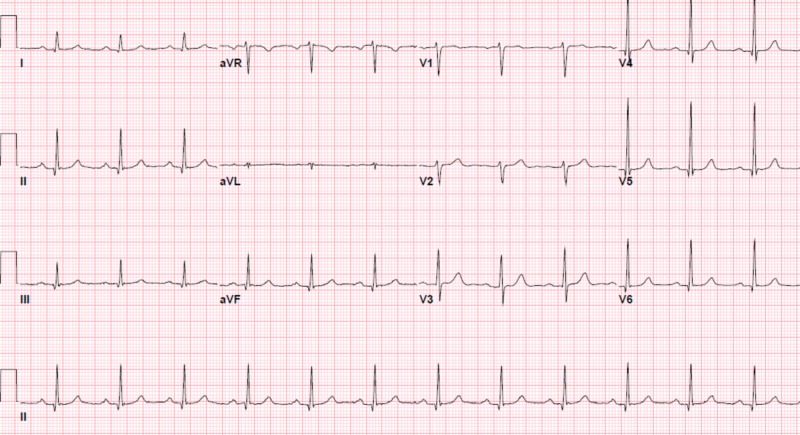
Electrocardiogram (EKG) at presentation showing a normal sinus rhythm

A previously healthy 61-year-old gentleman, with no prior risk factors for coronary artery disease, presented to the hospital with posterior chest pain after slipping on ice. Upon admission, he was found to have a hemoglobin of 8.7 g/dl and computed tomography (CT) scan of the chest and abdomen revealed a splenic hematoma. An electrocardiogram (EKG) and troponin level were normal at the time (Figure 1). He was discharged on pain medications after a subsequent workup was found to be unremarkable.

**Figure 2:**
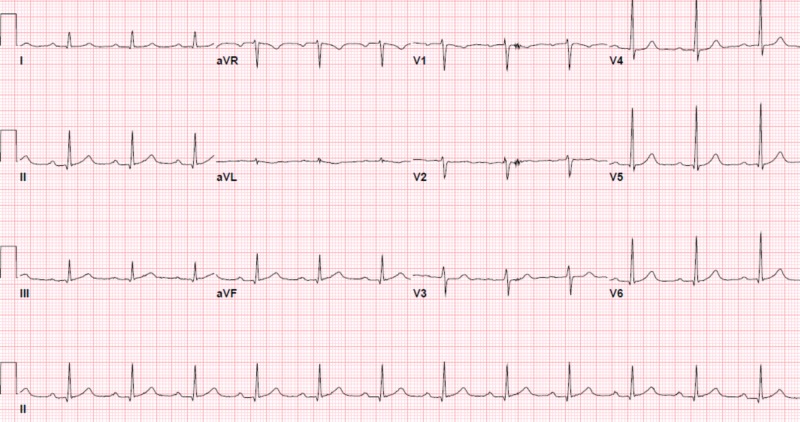
First-degree heart block with a PR interval of 228 ms.

The patient returned to the hospital two weeks later with dyspneic spells and dizziness. He was found to have an elevated troponin level of 0.049 ug/L that peaked at 3 ug/L (normal < 0.03 ug/l) over the next 15 hours. A hemoglobin level was stable at 8 g/dL, and thyroid stimulating hormone and B-type natriuretic peptide were noted to be normal. There was no acute process seen on cardiopulmonary imaging. Initial EKG showed a first-degree heart block (Figure 2) during this presentation. Transthoracic echocardiography (TTE) showed an ejection fraction of 60% with no valvular or wall motion abnormalities.

**Figure 3:**
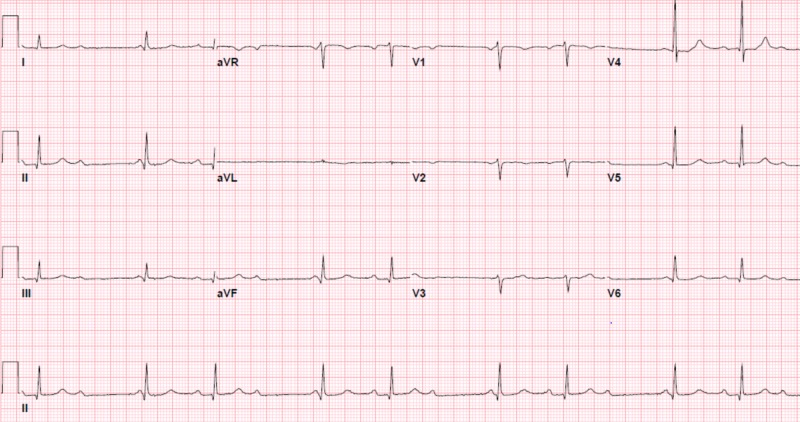
The following electrocardiogram three hours later had progressed to a second degree (type 1) heart block

**Figure 4:**
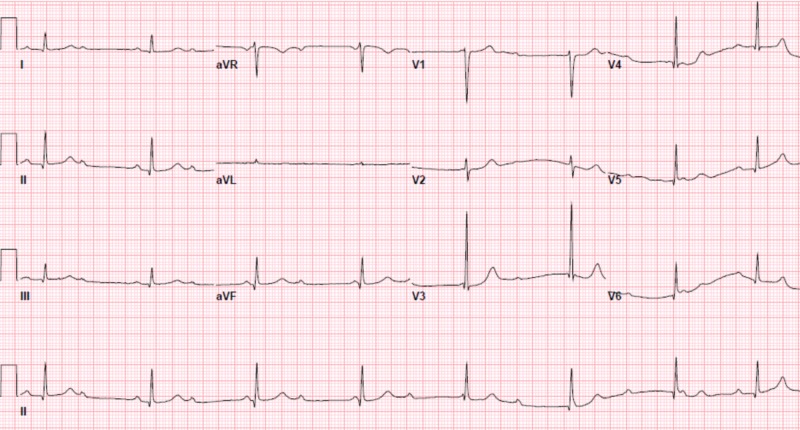
A subsequent electrocardiogram six hours later showed a complete (third-degree) heart block

A repeat EKG three hours later demonstrated progression to second-degree (Mobitz type 1) atrioventricular heart block (AVB) (Figure 3). A radionuclide stress test was consistent with a small and fixed defect of mild severity in the mid-anteroseptal and apical anterior location, consistent with myocardial infarction. Persistence of his presenting symptoms six hours later necessitated another EKG, which showed a complete heart block (Figure 4). This was managed urgently with a successful dual-chamber pacemaker, following which his symptoms resolved and his troponin levels normalized. He was discharged home on the following day.

## Discussion

Myocardial infarction (MI) is a known consequence of BCI causing cardiac contusion [6]. Just like an MI, the complications following the initial event may occur immediately or may take time to develop [7]. The nature of these complications varies from structural problems, such as valvular pathologies, to free wall rupture to arrhythmias and conduction defects. Unlike an MI, myocardial contusion does not have clear diagnostic criteria, which is worrisome as a lot of these cases need continued monitoring despite a normal initial workup [8].

Our case demonstrated a rapidly progressive first-degree heart block that presented two weeks after a fall. This quickly developed to potentially fatal complete heart block within a few hours, warranting pacemaker implantation. This is alarming as our patient had a normal EKG and troponin level after the trauma [9]. It was only the clinical acumen of the medical staff that led to prompt recognition and earlier management of the CHB.

High-degree AV block has been reported in both human and animal studies following BCI [10-12]. In a systematic review done by Ali et al. [3], 50% of patients reviewed in the literature had a recurrence or permanent CHB requiring pacemaker implantation and CHB secondary to BCI which was associated with a 20% mortality in the post-traumatic period. We strongly feel that even this high percentage of mortality is an underestimation as the long-term sequelae of BCI with delayed complications often are under-recognized [13]. Our patient developed a fatal heart block in the recovery period which could have easily been missed. The pathophysiology of this phenomenon is claimed to be secondary to necrosis, inflammation, or fibrotic changes in the subendocardial tissue which can progress and cause gradual development of complications from a blunt injury to the myocardium [14].

## Conclusions

In patients presenting with blunt cardiac trauma, it is important to recognize a myocardial injury, both in the acute phase and after discharge from the hospital. Closer monitoring and patient education are required for low-risk and high-risk patients as, even in the presence of normal initial cardiac workup, there remains a possibility of fatal arrhythmias and heart blocks during the myocardial recovery period. Robust diagnostic and surveillance guidelines in patients presenting with cardiac contusion following blunt cardiac injury are yet to be developed.

## References

[1] Maenza RL, Seaberg D, D'Amico F (1996). A meta-analysis of blunt cardiac trauma: ending myocardial confusion. Am J Emerg Med.

[2] Plautz CU, Perron AD, Brady WJ (2005). Electrocardiographic ST-segment elevation in the trauma patient: acute myocardial infarction vs myocardial contusion. Am J Emerg Med.

[3] Ali H, Furlanello F, Lupo P (2017). Clinical and electrocardiographic features of complete heart block after blunt cardiac injury: A systematic review of the literature. Heart Rhythm.

[4] Li W, Zhang L, Liang Y, Tong F, Zhou Y (2017). Sudden death due to the atrioventricular node contusion: three cases report. Medicine (Baltimore).

[5] Morsy M, Efeovbokhan N, Jha SK (2015). Complete heart block and asystole following blunt cardiac trauma. J Community Hosp Intern Med Perspect.

[6] Sybrandy KC, Cramer MJ, Burgersdijk C (2003). Diagnosing cardiac contusion: old wisdom and new insights. Heart.

[7] Liedtke AJ, DeMuth WE Jr (1973). Nonpenetrating cardiac injuries: a collective review. Am Heart J.

[8] Aykan AC, Oguz AE, Yildiz M, Özkan M (2013). Complete atrioventricular block associated with non-penetrating cardiac trauma in a 40-year-old man. J Emerg Med.

[9] Alborzi Z, Zangouri V, Paydar S (2016). Diagnosing myocardial contusion after blunt chest trauma. J Tehran Heart Cent.

[10] Soud M, Alrifai A, Kabach A, Fanari Z, Alraies MC (2018). Trauma-induced conduction disturbances. Ochsner J.

[11] Link MS, Wang PJ, Pandian NG (1998). An experimental model of sudden death due to low-energy chest-wall impact (commotio cordis). N Engl J Med.

[12] Maron BJ, Estes NAM (2010). Commotio cordis. N Engl J Med.

[13] Williams JC, Elkington WC (2008). Slow progressing cardiac complications--a case report. J Chiropr Med.

[14] Hamilton WJ (1976). Textbook of Human Anatomy, Vol 4. CV Mosby Co.

